# The Effect of N-Terminal Cyclization on the Function of the HIV Entry Inhibitor 5P12-RANTES

**DOI:** 10.3390/ijms18071575

**Published:** 2017-07-20

**Authors:** Anna F. Nguyen, Megan S. Schill, Mike Jian, Patricia J. LiWang

**Affiliations:** Molecular Cell Biology and the Health Sciences Research Institute, University of California Merced, 5200 North Lake Rd., Merced, CA 95343, USA; aankirskaia@ucmerced.edu (A.F.N.); mschill@caltech.edu (M.S.S.); jian.mike2@gmail.com (M.J.)

**Keywords:** 5P12-RANTES, Chemokine (C-C Motif) Ligand 5 (CCL5), HIV entry inhibition, N-terminal cyclization, chemokine

## Abstract

Despite effective treatment for those living with Human Immunodeficiency Virus (HIV), there are still two million new infections each year. Protein-based HIV entry inhibitors, being highly effective and specific, could be used to protect people from initial infection. One of the most promising of these for clinical use is 5P12-RANTES, a variant of the chemokine RANTES/CCL5. The N-terminal amino acid of 5P12-RANTES is glutamine (Gln; called Q0), a residue that is prone to spontaneous cyclization when at the N-terminus of a protein. It is not known how this cyclization affects the potency of the inhibitor or whether cyclization is necessary for the function of the protein, although the N-terminal region of RANTES has been shown to be critical for receptor interactions, with even small changes having a large effect. We have studied the kinetics of cyclization of 5P12-RANTES as well as N-terminal variations of the protein that either produce an identical cyclized terminus (Glu0) or that cannot similarly cyclize (Asn0, Phe0, Ile0, and Leu0). We find that the half life for N-terminal cyclization of Gln is roughly 20 h at pH 7.3 at 37 °C. However, our results show that cyclization is not necessary for the potency of this protein and that several replacement terminal amino acids produce nearly-equally potent HIV inhibitors while remaining CC chemokine receptor 5 (CCR5) antagonists. This work has ramifications for the production of active 5P12-RANTES for use in the clinic, while also opening the possibility of developing other inhibitors by varying the N-terminus of the protein.

## 1. Introduction

HIV, the virus that causes acquired immune deficiency syndrome (AIDS), infects about 2 million people each year, mostly throughout the developing world [1]. While effective treatments are available, particularly in economically advanced countries, there is no cure, and prevention remains a critical issue. A major strategy to prevent HIV infection is the development of microbicides, substances that could be used topically to prevent the sexual spread of HIV. Ideally, a microbicide would be active and remain functional in both the vaginal compartment (pH 4.0–4.5) [2] and the rectal compartment (pH 7) [3].

One of the most promising proteins under consideration for use as a microbicide is 5P12-RANTES. This protein was developed using random mutagenesis at the N-terminus of the chemokine RANTES (also called Chemokine (C-C Motif) Ligand 5 (CCL5)) [4], itself a weak HIV inhibitor. 5P12-RANTES binds tightly to the chemokine receptor CCR5 (CC chemokine receptor 5), which is the major co-receptor used by HIV to gain entry to the cell during initial infection [5,6]. 5P12-RANTES has also been shown to be stable at biological temperatures and in the presence of relevant bodily fluids [7], and it was fully protective as a topical microbicide when tested in macaques [8]. More recently, it has been reported as being under clinical development [9].

The N-terminal region of chemokines are known to be critical for function, with slight alterations or truncations known to cause significant differences in their interaction with their respective receptors [10,11,12,13]. For example, the simple addition of an N-terminal methionine caused RANTES/CCL5 to act as a CCR5 antagonist rather than an agonist [10], and truncation of the N-terminal residues of chemokines likewise led to proteins with the ability to bind but not activate CCR5 [12,14]. In developing chemokines as HIV inhibitors, chemically modifying the N-terminus of RANTES was found to be particularly effective. For example, one potent analog was AOP-RANTES, in which an organic extension replaced the first residue of RANTES [15]. This modified protein was not only a potent HIV inhibitor, but it also prevented chemotaxis of human monocytes; this is a valuable property because it is important to avoid bringing immune cells to a site where they could potentially be infected with HIV [16]. Researchers then used this design as a scaffold and tested various synthetic modifications to the N-terminus, resulting in the discovery of the even more potent PSC-RANTES; both AOP-RANTES and PSC-RANTES induce internalization of the CCR5 receptor [17,18]. However, neither of these variants could be produced recombinantly. To circumvent this issue, Hartley et al. developed a random mutagenesis/phage display technique with changes to the N-terminal resides of RANTES that led first to the production of P2-RANTES [13] and then to the discovery of 5P12-RANTES [4]. The mutations that produced 5P12-RANTES were focused on just the first ten amino acids in RANTES and showed over two orders of magnitude variation in inhibitory potency [4].

**Figure 1:**
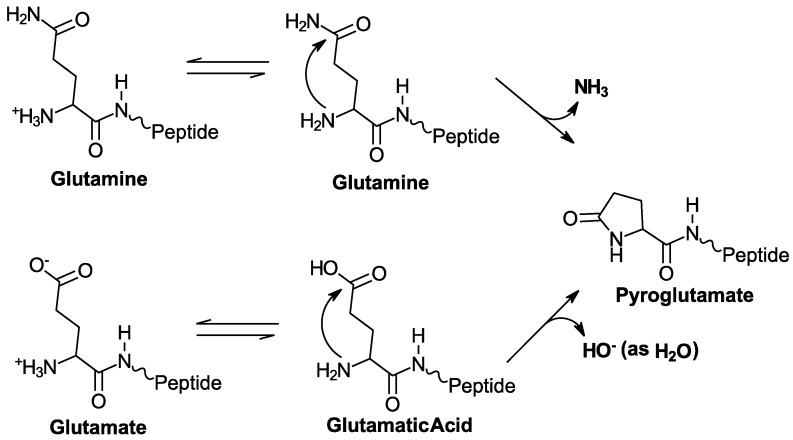
Cyclization reactions of N-terminal glutamine and glutamate residues in a polypeptide chain. Conditions such as pH are important factors in the rate of cyclization; low pH leads to a higher proportion of a good leaving group, while high pH leads to better nucleophilicity in the attacking amino group [22,26].

Interestingly, 5P12-RANTES as well as the other resultant inhibitors carried forward from the study, such as 5P14-RANTES and 6P4-RANTES, have a glutamine as the first amino acid (termed “Q0” because this is an additional amino acid added to the wild type RANTES sequence). N-terminal glutamines are able to chemically cyclize [19,20,21] (Figure 1), leading to possible heterogeneity in the protein product with two forms (cyclized and uncyclized) that are significantly different in charge and other chemical properties. This has also been a topic of significance in the development of antibodies as therapeutics, where N-terminal Gln and Glu are both common [19,22,23,24]. The increasing use of antibodies as therapeutics has led to several reports about the importance of cyclization of both Gln and Glu at the N-terminus of these and other clinically-relevant proteins [20,23,24,25].

While it has been recognized that 5P12-RANTES is likely to undergo N-terminal cyclization, the functional impact of this modification at such a critical region of the protein, as well as the details of the kinetics of cyclization, have not yet been reported [4,9,27]. Such information is critical both from the standpoint of understanding the mechanism of HIV inhibition and from the standpoint of identifying all forms of a molecule that may enter the clinic.

Here, we report a study on the N-terminal cyclization of 5P12-RANTES, including the effect of cyclization on CCR5 receptor function and HIV inhibition in two representative strains, as well as the rate of cyclization under various conditions. We also show the effect of mutating the N-terminal “Q0” to other amino acids, such as the residue glutamate, which cyclizes to form an identical molecule to “wild type” cyclized 5P12-RANTES.

## 2. Results

### 2.1. 5P12-RANTES N-Terminal Cyclization

**Figure 2:**
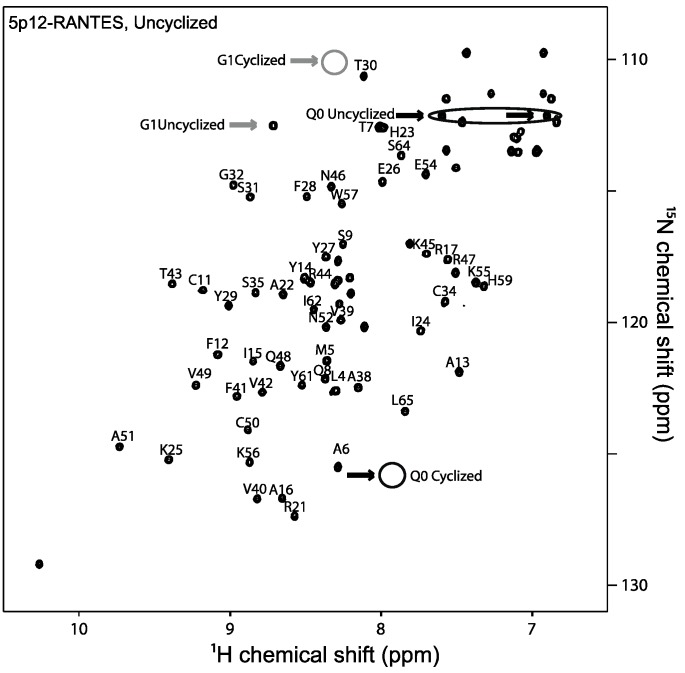
Heteronuclear single quantum coherence (HSQC) nuclear magnetic resonance (NMR) spectrum of ^15^N-labeled 5P12-RANTES directly after dissolution in pH 2.8 20 mM sodium phosphate buffer at 25 °C. Little or no cyclization is observed at this time. Cyclization of Q0 results in a shift of the G1 peak (labeled, grey arrows; cyclized position circled) which can be used to quantify the amount of cyclized 5P12-RANTES in solution. Cyclization also results in loss of Q0 side chain amide peaks (black arrows, circled) and appearance of cyclized Q0 lactam peak (black arrow, circle). Chemical shift assignments from Wiktor et al. [28]; no assignments are shown for region near E66, where these authors used a variant. Also not shown are side chain assignments for W57 and Asn/Gln (except for the relevant N-terminal side chain amide). Percent cyclization was determined by peak height at lower contour level than shown.

^15^N labeled 5P12-RANTES was produced from *E. coli* with an N-terminal fusion partner to disallow N-terminal cyclization of Q0 for most of the purification process. Cleavage of the fusion tag by enterokinase was carried out at pH 7.4 at 4 °C followed by reversed-phase chromatography and lyophilization of the pure protein so that it could be stored as dry powder to inhibit cyclization. The purified 5P12-RANTES was solubilized at pH 2.8 for Nuclear Magnetic Resonance (NMR) spectroscopy, where it was observed that less than 5% of the protein had undergone N-terminal cyclization during the purification process (Figure 2).

**Figure 3:**
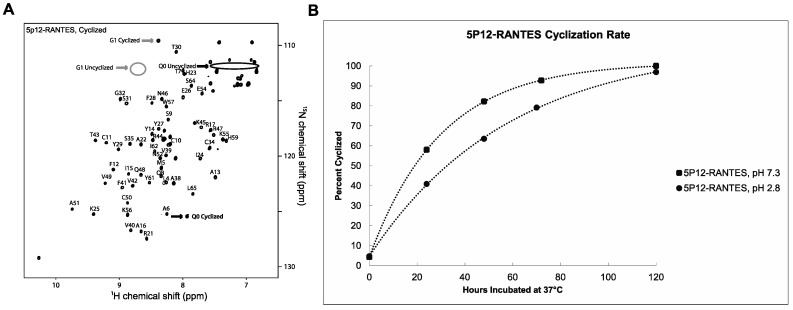
5P12-RANTES cyclization. (**A**) HSQC spectrum of cyclized ^15^N-labeled 5P12-RANTES after being incubated at 37 °C for 5 days at pH 2.8. NMR was performed in 20 mM sodium phosphate at pH 2.8, 25 °C. Cyclization results in a shift of the G1 residue (grey arrows; G1 resonances denoted by gray arrows and circles), as well as an appearance of the N-terminal pyroglutamate residue (black arrow; Q0 resonances denoted with black circles and arrows) as well as loss of Q0 amide side chain peaks (black arrow, circled). Assignments are not shown for certain areas as described in Figure 2. (**B**) Cyclization over time of 5P12-RANTES at pH = 7.3 and pH = 2.8, incubated at 37 °C. Amount of cyclization was determined by obtaining peak heights of the amide of G1 when the N-terminus of the protein (Gln0) was cyclized and uncyclized using NMRPipe, and dividing the cyclized peak height by the total of all G1 (cyclized and uncyclized) peak heights.

The cyclization rate of the N-terminal Gln of 5P12-RANTES at 37 °C was monitored by NMR at pH 7.3 and at pH 2.8 as shown in Figure 3B. A clear indication of cyclization at the N-terminal “Q0” position is the peak position of the backbone amide of glycine 1. This peak shows a clear shift from 8.7 ppm (^1^H) and 112.5 ppm (^15^N) to 8.4 ppm (^1^H) and 109.5 ppm (^15^N) as its neighboring side chain cyclizes [9] (Figure 2 and Figure 3A). Concomitantly, the lactam peak of the cyclized pyroglutamate (derived from Q0) is observed to grow in at 7.9 ppm (^1^H) and 125.5 ppm (^15^N) upon cyclization. At pH 7.3 these peaks are not discernable in an HSQC spectrum, likely due to faster amide exchange. Therefore NMR analysis for the pH 7.3 incubation was carried out at pH 2.8.

In order to monitor cyclization at pH 7.3, lyophilized powder of 5P12-RANTES was dissolved at pH 7.3, incubated at 37 °C, and aliquots were removed at various time intervals. These aliquots were lyophilized to halt further cyclization. Then immediately prior to measurement by NMR, each dry aliquot was dissolved in pH = 2.8 buffer. In this way, incubation occurred at neutral pH, but spectroscopy occurred at low pH where key peaks were visible in the spectrum, chemokine solubility is optimal, and on a time scale that would not allow significant further cyclization.

As shown in Figure 3B, Q0 cyclization of 5P12-RANTES at pH 7.3 (37 °C) exhibits a half life of about 20 h. When cyclization is measured with constant incubation at pH 2.8 (37 °C), the half life is about 33 h, with cyclization essentially complete at 120 h.

### 2.2. 5P12-Q0E Also Cyclizes to Produce Mature 5P12-RANTES

To further investigate the properties of the N-terminus of 5P12-RANTES, we produced 5P12-RANTES-Q0E, in which the Gln is replaced by the amino acid Glutamate, which is also capable of cyclizing when placed at the amino terminus of a protein. While the cyclization is expected to be slower due to the poor leaving group on the Glu side chain, the product of cyclization should be identical to cyclized Q0. This variant allows further study of cyclization and also provides an alternate route to the presumed mature product i.e., fully cyclized 5P12-RANTES containing a pyroglutamate at the N-terminus.

**Figure 4:**
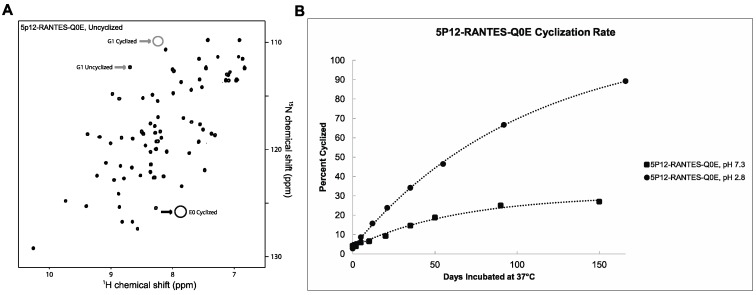
5P12-RANTES-Q0E. (**A**) HSQC spectrum of ^15^N-labeled 5P12-RANTES-Q0E. The spectrum is identical to that of 5P12-RANTES except for a slight G1 shift (grey arrows) and a loss of two side chain amide peaks corresponding to the Gln-0 NH_2_ group and no cyclized peak (black arrow, circled, lower right). Cyclization results in a shift of the G1 residue (labeled) which can be used to quantify the amount of cyclized 5P12-RANTES-Q0E in solution. Spectrum was measured in 20 mM sodium phosphate, pH 2.8, 25 °C. Percent cyclization was measured by peak height at lower contour levels than shown. (**B**) Cyclization over time of 5P12-RANTES-Q0E in pH = 7.3 and pH = 2.8, incubated at 37 °C. Spectrum at the 150 day time point shows folded protein, with some degradation (Figure S2).

Isotopically labeled ^15^N 5P12-RANTES-Q0E was produced with an N-terminal fusion protein to disallow cyclization until cleavage near the end of purification, in a similar manner as with 5P12-RANTES. Initial solubilization of purified, lyophilized protein followed by NMR revealed that less than 4% of this protein was cyclized during purification (Figure 4A). Incubation of ^15^N 5P12-RANTES-Q0E was carried out at pH 7.3 and pH 2.8 and monitored by NMR as described above. As shown in Figure 4B, the reaction to produce N-terminal pyroglutamate takes many days, with a half-life of greater than 150 days at pH 7.3 and a half-life of about 60 days at pH 2.8. This faster cyclization at low pH is expected due to improvement of the leaving group upon protonation. When cyclization is complete, the spectrum is identical to cyclized “wild type” 5P12-RANTES as expected (Figure S1).

### 2.3. High Potency HIV Inhibition Does Not Require N-terminal Cyclization or N-Terminal Glutamine 

To determine the relative importance of N-terminal cyclization on the anti-HIV potency of 5P12-RANTES, single round viral assays were carried out using two representative strains of HIV. Since these assays necessarily use mammalian cells, conditions are constrained to physiological pH (pH 7.3). In these assays, cells are aliquoted into 96 well plates and allowed to divide overnight. Various dilutions of inhibitor (freshly made from lyophilized powder and minimally cyclized) were incubated with the cells for 30 min. Virus was then added and the cells incubated overnight. Medium containing virus and inhibitor were removed the following day as medium was changed and cells were allowed to grow an additional two days with fresh medium. Infection was monitored with a standard β-galactosidase readout as described in Methods. The total time the inhibitor is in solution and able to cyclize is 20 h at pH 7.3 in this assay.

As shown in Table 1, “immature” 5P12-RANTES that was essentially uncyclized at the start of the assay gave an IC_50_ of 1.51 ± 0.10 nM for strain PVO.4 and 1.61 ± 0.12 nM for strain ZM53. Given the length of the assay, it is estimated that roughly 50% of the immature 5P12-RANTES did cyclize during the course of the assay. However, virus entry has been demonstrated to take place over the course of minutes [29], so a significant amount of virus-cell interaction could be expected before the inhibitor was significantly cyclized. “Matured” 5P12-RANTES (that was incubated for 36 h, pH 2.8 at 50 °C to allow full cyclization prior to use in the HIV assay) showed essentially equal potency, with an IC_50_ of 1.27 ± 0.10 nM for PVO.4 and 1.46 ± 0.15 nM for ZM53.

The mutant 5P12-Q0E showed somewhat poorer inhibition in its immature, uncyclized form, with an IC_50_ of 6.30 ± 0.14 nM for PVO.4. This variant has a negatively charged glutamate at position 0, a part of the protein that is presumed to interact with the receptor CCR5 in or near the hydrophobic membrane of the cell [30]. Given the half-life of cyclization described above, it is expected that during the assay, under 5% of this protein would be cyclized. When the matured, cyclized 5P12-Q0E (prepared as described in Methods) was used in these inhibition assays, its potency was essentially identical to wild type, cyclized 5P12-RANTES (Table 1), as expected for these two now-identical proteins.

As a control for uncyclized Q0 (“wild type”) 5P12-RANTES, we produced and purified 5P12-Q0N, in which the Gln is replaced by Asn. This amino acid is identical to Gln but has a shorter side chain so cannot similarly cyclize. As expected, the NMR spectrum of ^15^N 5P12-Q0N shows no indication of a cyclized N-terminus (Figure S3A). In HIV inhibition assays, this protein shows high potency, with an IC_50_ of 2.20 ± 0.33 nM against PVO.4 and 1.31 ± 0.19 against ZM53, essentially the same as for the wild type (Q0) protein, indicating that the presence of a cyclized pyroglutamate is not necessary for the anti-HIV activity of 5P12-RANTES.

Since both uncyclized and cyclized 5P12 variants were shown to be potent inhibitors of HIV, we made other mutations at the Q0 position of the protein to determine the effect of changes at this position on HIV inhibitory ability. For these experiments the Gln0 was changed to Phe, Ile, and Leu. Each of these variants was expressed and shown to be folded by NMR (Figure S3B–D); none of these side chains are expected to undergo significant chemical transformation upon incubation. As shown in Table 1, each variant was highly potent and very similar in activity to 5P12-RANTES, with IC_50_ values ranging from 1.21 ± 0.24 nM to 1.31 ± 0.13 nM (PVO.4) and 1.43 ± 0.11 nM to 1.84 ± 0.12 nM (ZM53).

### 2.4. The Effect of the N-Terminal Amino Acid and Its State of Cyclization on Activation of CC Chemokine Receptor 5 (CCR5)

**Figure 5:**
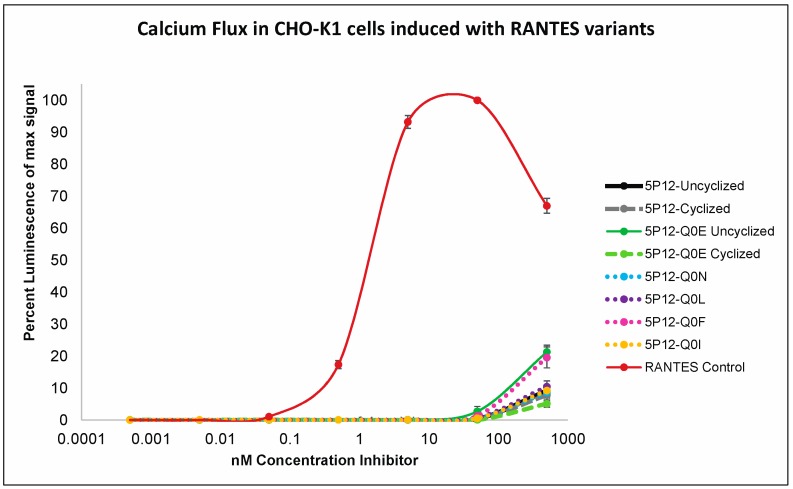
Calcium Flux Assay. CHO-K1 cells expressing CCR5 on their surface were incubated with various concentration of chemokine (either wild type RANTES/CCL5 or a 5P12-RANTES variant) and monitored for luminescence of aequorin upon calcium release. At very high “supraoptimal” concentrations, RANTES/CCL5 exhibits aggregation with alternate effects on receptor activation [31].

5P12-RANTES has been reported to be an antagonist of CCR5, not inducing calcium mobilization; lack of calcium mobilization is a property that is favorable (and likely necessary) in the context of therapeutic HIV inhibition. To determine whether the cyclization of Q0 or the placement of other amino acids at the N-terminus affect activation of CCR5, calcium flux assays were carried out on Chinese Hamster Ovary (CHO) cells expressing CCR5, G_α16_, and apo-aequorin, a calcium-responsive protein. As shown in Figure 5, neither N-terminally cyclized nor N-terminally uncyclized 5P12-RANTES (i.e., having Q0) caused a calcium flux until concentrations reached 500 nM, while wild type RANTES/CCL5 showed calcium release at low nM concentrations as expected. Further, the N-terminal variants of 5P12-RANTES (-Q0E cyclized and uncyclized, -Q0N, -Q0I, -Q0F, -Q0L) also exhibited no ability to cause the release of calcium until reaching 500 nM concentration (Figure 5). These results were further confirmed with a fluorescence assay using human HeLa-P5L cells, which showed similar results (Table S1).

## 3. Discussion

There are at least four highly potent classes of protein HIV entry inhibitors, each with great potential for clinical use in preventing viral infection. The first of these is broadly neutralizing antibodies [32], which bind gp120 or gp41 and show great promise, particularly when used in combination [33], but are generally produced in eukaryotic cells. [34,35] A second class of entry inhibitors includes lectins, which bind to glycosylation sites on HIV env and include the highly studied Griffithsin and Cyanovirin-N, both of which are being evaluated for potential clinical trials [36,37]. A third group are variations of peptides that are derived from HIV gp41, called fusion inhibitors [38], that are particularly effective in combination with other inhibitors [39,40].

Finally, in the fourth group, certain chemokine variants have been shown to inhibit entry of HIV due to their ability to bind the chemokine receptors that act as co-receptors for HIV. The most effective chemokine variants have been derived from the chemokine RANTES (Figure S4), originally by using synthetic modification at the N-terminus to produce PSC-RANTES, for example, which was shown to be effective in protecting macaques from infection [8,18]. Structural studies have shown that the N-terminus of the chemokine is likely to interact with the receptor at or near the cell membrane [41], and work on synthetic RANTES variants supported these results, showing that hydrophobic groups at the N-terminus were more effective at interacting with the CCR5 receptor than similar variants with hydrophilic modifications [18].

More recently, random mutagenesis/phage display was employed to select for RANTES N-terminal variants with enhanced ability to inhibit HIV. This work first led to P2-RANTES [13], which was used as a starting point for later selection of a series of even more highly potent, fully-recombinant RANTES variants [4]. This latter set of inhibitors included 5P12-RANTES, 5P14-RANTES, and 6P4-RANTES, each of which has slight differences in its 10 N-terminal amino acids, leading to differing ability to activate and/or internalize the CCR5 receptor, and again, providing evidence that the N-terminal region of the chemokine is critical in interacting with the receptor and affecting its conformational changes in the membrane. The most clinically promising of these inhibitors is generally considered to be 5P12-RANTES, due to its combination of high potency and inability to induce downstream signaling or internalization of the CCR5 receptor. Therefore, this inhibitor can inhibit HIV without activating the immune system or mediating an influx of immune cells (and by virtue of being a CCR5 antagonist, may actually reduce immune activity).

5P12-RANTES has been shown to have many properties necessary for use as a topical microbicide, including stability at elevated temperatures, a range of pH conditions, and within environments containing bodily fluids [7,42]. This inhibitor demonstrated the ability to prevent infection in macaques [8] and has shown effectiveness against many strains of HIV with little or no indication that HIV is able to mutate to lose sensitivity to it [43]. Therefore, this protein is a top candidate for a clinical HIV microbicide.

A critical issue for any protein moving to the clinic is a clear understanding of its function, as well as characterization of any variation in its chemical or structural composition. For instance, antibodies often have Glu or Gln as N-terminal residues, and the growing importance of antibodies as therapeutics has led to the study of these residues’ propensity to cyclize [19,22,23,24]. 5P12-RANTES has an N-terminal Gln (referred to as Q0), an amino acid that is known to cyclize when at this position in a protein [19,44], leading to an extra cyclization step during clinical-grade production [27]. However, a detailed study of the rate and functional effect of this cyclization in 5P12-RANTES has not been reported.

Wiktor et al. have provided NMR chemical shift assignments and the structure of a non-aggregating variant of 5P12-RANTES [9,28]. This group estimated a half-life for Q0 cyclization as approximately two days at pH 3.8, although the sample was refrigerated during part of this time, since the primary goal of the work was not to study N-terminal cyclization but rather to investigate the overall structure of the protein and its interactions with detergents.

We have shown here that the N-terminus of 5P12-RANTES cyclizes to form pyroglutamate with a half-life of roughly 20 h at pH 7.3 and 33 h at pH 2.8 (37 °C). This indicates that the protein, if purified without regard to cyclization, would still cyclize on the time scale of a typical HIV assay (20 h at pH 7.3), leaving inconclusive whether high potency is reserved for only the cyclized form. However, when Q0 is replaced with N, such cyclization is not observed, and this variant (5P12-Q0N) shows essentially identical HIV-inhibitory ability as 5P12-RANTES, indicating that a cyclized N-terminus is not important for the function of this inhibitor. Other hydrophobic N-terminal variants, -Q0I, -Q0F, -Q0L, also show very high potency, nearly equivalent to 5P12-RANTES (Table 1).

To further investigate the importance of the cyclized form of 5P12-RANTES, we made 5P12-Q0E, in which glutamate replaces glutamine at the N-terminus of the protein. Glutamate can also be expected to cyclize and form the same pyroglutamate as is formed by glutamine (Figure 1), although a poorer leaving group is expected to cause this reaction to be slower. Upon cyclization, this variant will yield a product that is identical to cyclized 5P12-RANTES.

It was observed that at 37 °C, 5P12-Q0E cyclization occurs with a half-life of well over 150 days at pH 7.3 (with still only 25% cyclization observed at 150 days) and roughly 60 days at pH 2.8. The fully cyclized version of the variant (prepared by prolonged incubation at higher temperatures) was tested against two strains of HIV and shown to be essentially identical in function to cyclized 5P12-RANTES containing the original Q0. Interestingly, the uncyclized 5P12-Q0E also showed good inhibitory properties despite the timescale of the assay not allowing significant cyclization and therefore leaving a negatively charged Glu at the terminus of the protein throughout the assay (Table 1).

These results clarify the conditions necessary to cyclize 5P12-RANTES if it were to be used clinically in a homogeneous form. They further show that not only is cyclization not necessary for anti-HIV activity, but also that a variety of amino acids at the N-terminus would be expected to provide protection against HIV infection. This allows a greater range of amino acid sequences as potential inhibitors, which could lead to flexibility in determining which sequence most easily results in production of the large amounts of protein required for clinical use.

In conclusion, 5P12-RANTES is a highly potent HIV inhibitor that could be used as a microbicide to prevent HIV infection. However, chemical cyclization of its N-terminal glutamine causes this protein to exist as a heterogeneous mixture when expressed recombinantly. We show that the protein is still a highly effective inhibitor of HIV in both its cyclized and uncyclized forms and have determined the rate of cyclization under various conditions, with relevance both for the manufacture and clinical use of this protein. We also show that several amino acids are suitable replacements for the N-terminal glutamine if changes in the sequence are necessary or desirable.

## 4. Materials and Methods

### 4.1. Protein Purification

In brief, the following method was used (with the exception of wild type RANTES/CCL5, which required some differences as noted): Plasmids were transformed into *Escherichia coli* BL21 (DE3) (Novagen) competent cells and expressed in minimal media with ^15^NH_4_Cl as the sole nitrogen source. Protein production was induced with the addition of isopropyl β-d-1-thiogalactopyranoside (IPTG) and incubated with shaking at 22 °C for 20 h (wild type RANTES/CCL5 was induced with IPTG followed by shaking at 37 °C for 6 h). The cell pellet was resuspended in 6 M guanidine hydrochloride, 200 mM NaCl, 10 mM benzamidine, and 50 mM Tris (pH 8.0) and was then disrupted by French press and then centrifuged at 27,000× *g* for 1 h. The soluble portion was loaded onto a nickel chelating column (Qiagen, Hilden, Germany) equilibrated with the resuspension buffer. Proteins were eluted from the column using a pH gradient with 6 M guanidine hydrochloride, 200 mM NaCl, and 60 mM NaOAc (pH 4) followed by addition of 10 mM β-mercaptoethanol with stirring for one hour at room temperature. The proteins were then refolded by dropwise addition to 10× volume of refolding buffer (550 mM L-Arginine Hydrochloride, 200 mM NaCl, 1 mM EDTA, 1 mM reduced glutathione (GSH), 0.1 mM oxidized glutathione (GSSG), 50 mM Tris, pH 8), and then allowed to stir overnight at 4 °C. The solution was dialyzed twice into 4 liters 200 mM NaCl, 2 mM CaCl_2_, 20 m Tris, pH 7.4 buffer at 4 °C (into 4 L of 200 mM NaCl, 20 mM Tris pH 8 for wild type RANTES).

To cleave fusion tags from the purified protein, the samples were incubated for 24 h with 650 nM of the protease enterokinase (5P12-RANTES-Q0E required 3 days at a 2 μM; 100 nM ULP-1/SUMO protease was used to cleave wild type RANTES/CCL5; protease purification described below) The protein solution was then centrifuged to remove precipitated material and added onto a second nickel chelating column (Qiagen, Hilden, Germany), with unbound eluate (containing the cleaved 5P12-RANTES or its variants) being collected. (This second nickel column is not necessary for wild type RANTES/CCL5 purification). The samples were then dialyzed and purified on a C_4_ reversed-phase chromatography column (Vydac, Hesperia, CA, USA), using an acetonitrile gradient. Wild type RANTES/CCL5 was also purified with the C_4_ column, allowing separation from the SUMO tag. Overall, samples were near neutral pH for about a day during proteolytic cleavage (3 days for 5P12-Q0E due to slow cleavage) and a day for dialysis to prepare conditions for the C4 column, during which time N-terminal cyclization was possible. However, dialysis was carried out at 4 °C to minimize this reaction, and as noted in Results, little cyclization was observed after this point. The fractions were analyzed on an SDS-PAGE gel to confirm purity and then lyophilized in a Labconco freeze-dry system.

N-terminally cyclized proteins used for functional assays (below) were prepared as follows: purified and lyophilized 5P12-RANTES was incubated for 36 h at 50 °C in 20 mM sodium phosphate buffer pH 2.8 to ensure 100% cyclization. 5P12-RANTES-Q0E was incubated for 50 days at 50 °C in 20 mM sodium phosphate buffer pH 2.8 to fully cyclize. Both samples were tested by NMR to confirm structural integrity and full cyclization (Figure S1).

The proteases used in these purifications were produced and purified in our laboratory as briefly described: Ubl-specific protease 1 or enterokinase protease were proteins were expressed in Luria Broth (LB) medium using a pET-28b vector and the cells were collected and French pressed. The ULP1 protease from the supernatant was purified using a nickel chelating column [45]. The enterokinase was found in the inclusion body and resuspended in 6 M guanidinium chloride buffer before being purified using a nickel chelating column. Enterokinase was then dialyzed in buffer to allow for refolding and tested for activity through self-cleavage of the fusion tag (manuscript in preparation).

### 4.2. Obtaining Rates of 5P12-RANTES and 5P12-RANTES-Q0E Cyclization

Samples incubated at pH 2.8 were prepared by adding ^15^N-labeled lyophilized protein (5P12-RANTES or 5P12-RANTES-Q0E) to 20 mM sodium phosphate buffer with 10% D_2_O and 5 µM DSS, resulting in a final pH = 2.8. Samples were placed into Shigemi NMR tubes (Shigemi, Inc., Allison Park, PA, USA) and incubated at 37 °C. NMR spectra were periodically measured at 37 °C for these samples (see below).

Samples at pH = 7.3 (the same pH as the pseudoviral assay medium) were prepared by adding ^15^N-labeled lyophilized protein (5P12-RANTES or 5P12-RANTES-Q0E) to 5 mM Sodium Phosphate buffer with 0.02% sodium azide with a final pH of pH 7.3. Proteins were incubated at 37 °C, and samples were removed at selected time points, flash-frozen using liquid nitrogen, and lyophilized. Samples were then dissolved in 20 mM Phosphate buffer, 5% D_2_O, and 5 µM DSS with a final pH = 2.8, and then measured by NMR at 25 °C (see below). All measurements for the pH of protein samples were taken with a micro electrode (Thermo Scientific/Orion).

### 4.3. NMR Spectroscopy

^15^N-labeled lyophilized proteins were added into 20 mM Sodium Phosphate buffer with 10% D_2_O and 5 µM 2,2-dimethyl-2-silapentane-5-sulfonic acid (DSS), with a final pH = 2.8 for all samples. Concentration of protein ranged from 30 uM to 80 uM. Sample preparation for cyclization time points are further described above. All NMR data were acquired on a four-channel 600 MHz Bruker Avance III spectrometer equipped with a GRASP II gradient accessory and a TCI cryoprobe with an actively shielded Z-gradient coil. Spectra were measured at 25 °C or 37 °C. The chemical shift was referenced relative to internal DSS [46]. The data were processed using NmrPipe [47] and analyzed using PIPP [48]. For HSQC spectra, sweep width = 8474.576 (^1^H) and 1766.784 Hz (^15^N), with 1280 points in ^1^H and 128 * (256 total) points in ^15^N.

### 4.4. Quantifying N-Terminal Cyclization

N-terminal cyclization of 5P12-RANTES or 5P12-RANTES-Q0E results in the appearance of an N-terminal lactam peak “Gln0” (Figure 1 and Figure 2), as well as a shift in the Gly1 backbone amide peak. [9,28] (Figure 2 and Figure 3A). Peak height comparison of the Gly1 amide when the N-terminus of the protein is cyclized versus uncyclized was used to estimate the percent of the protein cyclized in the sample as follows. NmrPipe was used to obtain peak heights for the Gly1 amide. The percent cyclization was obtained by dividing the height of the Gly1 peak when the N-term was cyclized by the sum of the heights of both cyclized and uncyclized Gly1 peaks in a spectrum. Similar values were obtained when using the peak height of the cyclized Q0 peak to measure the cyclized amount. Using peak volumes rather than height did not appreciably change the result.

### 4.5. Cell Lines and Viruses

HeLa-TZM-bl cells were obtained through the AIDS Research and Reference Reagent Program, Division of AIDS, National Institute of Allergy and Infectious Diseases (NIAID), National Institutes of Health; the HeLa-TZM-bl cell line was from John C. Kappes, Xiaoyun Wu, and Tranzyme Inc. 293FT cells were originally obtained from Invitrogen; CHO-K1 cells stably expressing CCR5, G_α16_, and apo-aequorin were a kind gift from Marc Parmentier from the Institute of Interdisciplinary Research of the Free University of Brussels (ULB) Medical School, Brussels, Belgium. HeLa-P5L Cells (a cell line stably expressing human receptor CD4 and CCR5) was a kind gift from M. Alizon (Cochin Institute, Paris, France) [49].

Viral plasmids used to create pseudovirus, including HIV-1 PVO clone 4 (SVPB11), ZM53M.PB12 (SVPC11), and pSG3^Δenv^, were obtained from the AIDS Research and Reference Reagent Program, Division of AIDS, NIAID, National Institutes of Health; PVO, clone 4 (SVPB11) was from David Montefiori and Feng Gao [50]; ZM53M.PB12, SVPC11 was from Cynthia A. Derdeyn and Eric Hunter [51]; plasmid pSG3^Δenv^ was obtained from John C. Kappes and Xiaoyun Wu [51,52,53].

### 4.6. Single Round Pseudovirus Production

293FT cells were co-transfected with the pSG3^Δenv^ plasmid and an envelope plasmid using the XTreme Gene Transfection Reagent (Roche/Sigma-Aldrich, St. Louis, MO, USA). 48 h post-transfection, the supernatant was collected, centrifuged, and filtered with a 0.45-μm syringe filter. The viral stocks were then stored at −80 °C.

### 4.7. Single-Round Pseudoviral Assay

HeLa-TZM-bl cells were grown in culture media (10% Fetal Bovine Serum (FBS), penicillin/streptomycin, 25 mM HEPES in Dulbecco’s Modified Eagle’s Medium (DMEM)). 10^4^ cells per well were seeded into a 96-well plate and allowed to incubate at 37 °C overnight. Media was then aspirated and replaced with 50 µL fresh media and allowed to incubate for 2 h. For preparation of inhibitor, a deep-well dish was made with varying concentrations of inhibitor diluted into phosphate-buffered saline (PBS) and mixed thoroughly. 20 µL of inhibitor from these dilutions (or 20 µL PBS for controls) were added to the TZM cell-containing wells, and the plate was rotated for 1 min to mix, before incubating for 30 min at 37 °C. 30 µL of single-round virus (described above) in media was then added (with non-viral media to the negative control) for a final volume of 100 µL/well and the plate was rotated for 1 min to mix. This “layering” method differs from other similar pseudoviral assays, where the inhibitor is mixed in-well. In our hands, mixing in-well leads to lower IC_50_ (apparent better inhibition), but current experiments use “layering” addition to more closely mimic conditions under which a topical microbicide may be used. After 20 h, media was aspirated and replaced with 100 µL/well of fresh media. After 48 more hours, the media was aspirated and the cells were lysed using 30 µL/well of 0.5% NP-40 in PBS. Lysed cells were incubated at room temperature for 25 min. Substrate was added (30 µL/well; 8 mM chlorophenol red-β-d-galactopyranoside, 10 mM β mercaptoethanol, 20 mM KCl in PBS). The absorbance signals at wavelengths 570 and 630 nm were measured, and the 570:630 ratio for each well was calculated. All inhibitors were tested in triplicate repeated three times. The data were plotted using Microsoft Excel, and the IC_50_ value was determined using a linear equation fitted between two data points surrounding 50% inhibition.

### 4.8. CCR5 Functional Assays in CHO-K1 Cells

The functional response of CCR5-expressing cells to RANTES and its variants was analyzed by measuring the luminescence of aequorin in response to release of Ca^2+^ as described previously [54]. Briefly, CHO-K1 cells were cultured in Ham’s F12 medium (Corning, Cellgro, Manassas, VA, USA) supplemented with 10% FBS (Life Technologies, Carlsbad, CA, USA), 100 units/mL penicillin, 100 µg/mL streptomycin, and 400 µg/mL G418 (Life technologies, Carlsbad, CA, USA), then removed from plates by incubating at 37 °C with versene (Gibco, Gaithersburg, MD, USA), and then adding 3× volume Ca^2+^- and Mg^2+^-free DMEM (GE Healthcare, Little Chalfont, UK) to gently resuspend. The cells were gently pelleted for 4 min at 600× *g* and then resuspended at a density of 5 × 10^6^ cells/mL using Ham’s F12 Medium supplemented with 10% FBS, penicillin/streptomycin, 25 mM HEPES. Cells were then incubated in the dark at room temperature, for 1 h with 5 µM coelenterazine H (Promega, Madison, WI, USA). Cells were then diluted five-fold using Ham’s F-12 medium, and 50 µL (50,000 cells) were added per well in a 96-well plate. 50 µL of each inhibitor diluted in Ham’s F-12 medium were added to each well at varying concentrations. Luminescence was measured in 30 s increments using an Orion II microplate luminometer (Berthold Techniques, Bad Wildbad , Germany).

### 4.9. CCR5 Activity in HeLa-P5L

The functional response of CCR5-expressing cells HeLa-P5L to RANTES and its variants was analyzed by measuring the fluorescence using Fluo-4 dye (Thermo Scientific, Waltham, MA, USA) according to the manufacturer’s suggested protocol. Briefly, HeLa-P5L cells were cultured in RPMI-1640 (Invitrogen, Carlsbad, CA, USA) supplemented with 10% FBS, and 100 units of penicillin and 0.1 mg/mL streptomycin. The expression of CCR5 was selectively expressed by adding zeocin (Invitrogen, Carlsbad, CA, USA) at 0.5 mg/mL. Cells were then removed from plates by incubating at 37 °C with versene solution (Gibco, Gaithersburg, MD, USA) and then gently resuspended with culture media. The cells were gently pelleted for 4 min at 600× *g* and then resuspended at a density of 1 × 10^6^ cells/mL and 100 µL was plated onto 96 well plates with black polystyrene wells and a flat, micro-clear bottom (Greiner CELLSTAR). After a 15 h incubation at 37 °C, wells were washed three times with sterile Hank’s buffered salt solution (HBSS), 137 mM NaCl, 5.4 mM KCl, 0.25 mM Na_2_HPO_4_, 0.44 mM KH_2_PO_4_, 1.3 mM CaCl_2_, 1.0 mM MgSO_4_, 1.0 mM MgCl_2_, 10 mM glucose, 10 mM HEPES, with pH adjusted to 7.4 using 1N NaOH. 50 µL of 2 µM Fluo-4-AM Ester (Invitrogen, Carlsbad, CA, USA) in HBSS was added. The plate was incubated in the dark at room temperature for 30 min, then washed three times with HBSS supplemented with 2 mM Probenecid (Tocris, Bristol, UK), then left to incubate with 80 µL/well of HBSS with 2 mM Probenecid for 30 min at 37 °C. Fluorescence was measured after addition of 20 µL of chemokine variants in HBSS for a final chemokine concentration of 300 nM/well. Fluorescence reading was done on a Cytation 5 Cell Imaging Multi-Mode Reader (Biotek, Winooski, VT, USA) with an absorption/emission at 494/516 nm at 35 s, with wild type RANTES/CCL5 as a positive control and HBSS containing no inhibitor as a negative control.

## Figures and Tables

**Table 1:** IC_50_ values of 5P12-RANTES variants (in nM) against a Clade B HIV viral strain (PVO.4) or Clade C viral strain (ZM53) in single-round pseudoviral infection assays. Assays were performed in triplicate and repeated three times.

IC_50_ Values of 5P12 Variants		
5P12 Variant	PVO.4 (nM)	ZM53 (nM)
5P12-RANTES Uncyclized	1.51 ± 0.10	1.61 ± 0.12
5P12-RANTES Cyclized	1.27 ± 0.10	1.46 ± 0.15
5P12-RANTES-Q0E Uncyclized	6.30 ± 0.14	4.04 ± 0.29
5P12-RANTES-Q0E Cyclized	1.09 ± 0.06	1.23 ± 0.09
5P12-RANTES-Q0N	2.20 ± 0.33	1.31 ± 0.19
5P12-RANTES-Q0I	1.21 ± 0.24	1.43 ± 0.11
5P12-RANTES-Q0F	1.26 ± 0.19	1.84 ± 0.12
5P12-RANTES-Q0L	1.31 ± 0.12	1.45 ± 0.15

## Supplementary Materials

Supplementary materials can be found at www.mdpi.com/1422-0067/18/7/1575/s1.

## Abbreviations

HIV	Human Immunodeficiency Virus
AIDS	Acquired Immune Deficiency Syndrome
CCL5	Chemokine (C-C Motif) Ligand 5; also called RANTES
RANTES	Regulated on Activation, Normal T-cell Expressed and Secreted; also called CCL5
Gln/Q	Glutamine
Glu/E	Glutamate
Asn/N	Asparagine
Phe/F	Phenylalanine
Ile/I	Isoleucine
DMEM	Dulbecco’s Modified Eagle’s Medium
NMR	Nuclear Magnetic Resonance
HBSS	Hank’s Buffered Salt Solution
FBS	Fetal Bovine Serum
HSQC	Heteronuclear Single Quantum Coherence
CHO	Chinese Hamster Ovary
IPTG	Isopropyl β-d-1-thiogalactopyranoside
TFA	Trifluoroacetic Acid

## References

[1] Global Report: Unaids UNAIDS Report on the Global AIDS Epidemic 2013 http://www.unaids.org/sites/default/files/media_asset/UNAIDS_Global_Report_2013_en_1.pdf.

[2] Boskey E.R., Telsch K.M., Whaley K.J., Moench T.R., Cone R.A. (1999). Acid production by vaginal flora in vitro is consistent with the rate and extent of vaginal acidification. Infect. Immun..

[3] Evans D.F., Pye G., Bramley R., Clark A.G., Dyson T.J., Hardcastle J.D. (1988). Measurement of gastrointestinal pH profiles in normal ambulant human subjects. Gut.

[4] Gaertner H., Cerini F., Escola J.-M., Kuenzi G., Melotti A., Offord R., Ne Rossitto-Borlat I., Nedellec R., Salkowitz J., Gorochov G. (2008). Highly potent, fully recombinant anti-HIV chemokines: Reengineering a low-cost microbicide. Proc. Natl. Acad. Sci. USA.

[5] Suresh P., Wanchu A. (2006). Chemokines and chemokine receptors in HIV infection: Role in pathogenesis and therapeutics. J. Postgrad. Med..

[6] Dragic T., Litwin V., Allaway G.P., Martin S.R., Huang Y., Nagashima K.A., Cayanan C., Maddon P.J., Koup R.A., Moore J.P. (1996). HIV-1 entry into CD4+ cells is mediated by the chemokine receptor CC-CKR-5. Nature.

[7] Cerini F., Landay A., Gichinga C., Lederman M.M., Flyckt R., Starks D., Offord R.E., Xois F., Gal L., Hartley O. (2008). Chemokine Analogues Show Suitable Stability for Development as Microbicides. J. Acquir. Immune Defic. Syndr..

[8] Veazey R.S., Ling B., Green L.C., Ribka E.P., Lifson J.D., Piatak M., Lederman M.M., Mosier D., Offord R., Hartley O. (2009). Topically Applied Recombinant Chemokine Analogues Fully Protect Macaques from Vaginal Simian- Human Immunodeficiency Virus Challenge. J. Infect. Dis..

[9] Wiktor M., Hartley O., Grzesiek S. (2013). Characterization of structure, dynamics, and detergent interactions of the anti-HIV chemokine variant 5P12-RANTES. Biophys. J..

[10] Proudfoot A.E.I., Power C.A., Hoogewerf A.J., Montjovent M.O., Borlat F., Offord R.E., Wells T.N.C. (1996). Extension of recombinant human RANTES by the retention of the initiating methionine produces a potent antagonist. J. Biol. Chem..

[11] Laurence J.S., Blanpain C., De Leener A., Parmentier M., LiWang P.J. (2001). Importance of basic residues and quaternary structure in the function of MIP-1β: CCR5 binding and cell surface sugar interactions. Biochemistry.

[12] Laurence J.S., LiWang A.C., LiWang P.J. (1998). Effect of N-terminal truncation and solution conditions on chemokine dimer stability: Nuclear magnetic resonance structural analysis of macrophage inflammatory protein 1β mutants. Biochemistry.

[13] Hartley O., Dorgham K., Perez-Bercoff D., Cerini F., Heimann A., Gaertner H., Offord R.E., Pancino G., Debré P., Gorochov G. (2003). Human Immunodeficiency Virus Type 1 Entry Inhibitors Selected on Living Cells from a Library of Phage Chemokines. J. Virol..

[14] Arenzana-Seisdedos F., Virelizier J.-L., Rousset D., Clark-Lewis I., Loetscher P., Moser B., Baggiolini M. (1996). HIV blocked by chemokine antagonist. Nature.

[15] Simmons G., Clapham P.R., Picard L., Offord R.E., Rosenkilde M.M., Schwartz T.W., Buser R., Wells T.N., Proudfoot A.E., Cocchi F. (1997). Potent inhibition of HIV-1 infectivity in macrophages and lymphocytes by a novel CCR5 antagonist. Science.

[16] White G.E., Iqbal A.J., Greaves D.R. (2013). CC chemokine receptors and chronic inflammation--therapeutic opportunities and pharmacological challenges. Pharmacol. Rev..

[17] Pastore C., Picchio G.R., Galimi F., Fish R., Hartley O., Offord R.E., Mosier D.E. (2003). Two mechanisms for human immunodeficiency virus type 1 inhibition by N-terminal modifications of RANTES. Antimicrob. Agents Chemother..

[18] Hartley O., Gaertner H., Wilken J., Thompson D., Fish R., Ramos A., Pastore C., Dufour B., Cerini F., Melotti A. (2004). Medicinal chemistry applied to a synthetic protein: Development of highly potent HIV entry inhibitors. Proc. Natl. Acad. Sci. USA.

[19] Kumar M., Chatterjee A., Khedkar A.P., Kusumanchi M., Adhikary L. (2013). Mass spectrometric distinction of in-source and in-solution pyroglutamate and succinimide in proteins: A case study on rhG-CSF. J. Am. Soc. Mass Spectrom..

[20] Liu Y.D., Goetze A.M., Bass R.B., Flynn G.C. (2011). N-terminal glutamate to pyroglutamate conversion in vivo for human IgG2 antibodies. J. Biol. Chem..

[21] Tritsch G.L., Moore G.E. (1962). Spontaneous decomposition of glutamine in cell culture media. Exp. Cell Res..

[22] Yu L., Vizel A., Huff M.B., Young M., Remmele R.L., He B. (2006). Investigation of N-terminal glutamate cyclization of recombinant monoclonal antibody in formulation development. J. Pharm. Biomed. Anal..

[23] Chelius D., Jing K., Lueras A., Rehder D.S., Dillon T.M., Vizel A., Rajan R.S., Li T., Treuheit M.J., Bondarenko P.V. (2006). Formation of pyroglutamic acid from N-terminal glutamic acid in immunoglobulin gamma antibodies. Anal. Chem..

[24] Yan B., Valliere-Douglass J., Brady L., Steen S., Han M., Pace D., Elliott S., Yates Z., Han Y., Balland A. (2007). Analysis of post-translational modifications in recombinant monoclonal antibody IgG1 by reversed-phase liquid chromatography/mass spectrometry. J. Chromatogr. A.

[25] Kumar A., Bachhawat A.K. (2012). Pyroglutamic acid: Throwing light on a lightly studied metabolite. Curr. Sci..

[26] Dick L.W., Kim C., Qiu D., Cheng K.-C. (2007). Determination of the origin of the N-terminal pyro-glutamate variation in monoclonal antibodies using model peptides. Biotechnol. Bioeng..

[27] Cerini F., Gaertner H., Madden K., Tolstorukov I., Brown S., Laukens B., Callewaert N., Harner J.C., Oommen A.M., Harms J.T. (2016). A scalable low-cost cGMP process for clinical grade production of the HIV inhibitor 5P12-RANTES in Pichia pastoris. Protein Expr. Purif..

[28] Towards NMR Analysis of the HIV-1 Coreceptor CCR5 and Its Interaction with RANTES http://edoc.unibas.ch/30282/.

[29] Grewe C., Beck A., Gelderblom H.R. (1990). HIV: Early virus-cell interactions. J. Acquir. Immune Defic. Syndr..

[30] Allen S.J., Crown S.E., Handel T.M. (2007). Chemokine:Receptor Structure, Interactions, and Antagonism. Annu. Rev. Immunol..

[31] Appay V., Brown A., Cribbes S., Randle E., Czaplewski L.G. (1999). Aggregation of RANTES Is Responsible for Its Inflammatory Properties: Characterization of Nonaggregating, Noninflammatory RANTES Mutants. J. Biol. Chem..

[32] Burton D.R., Hangartner L. (2016). Broadly Neutralizing Antibodies to HIV and Their Role in Vaccine Design. Annu. Rev. Immunol..

[33] Klein F., Halper-Stromberg A., Horwitz J.A., Gruell H., Scheid J.F., Bournazos S., Mouquet H., Spatz L.A., Diskin R., Abadir A. (2012). HIV therapy by a combination of broadly neutralizing antibodies in humanized mice. Nature.

[34] Clark A.J., Gindin T., Zhang B., Wang L., Abel R., Murret C.S., Xu F., Bao A., Lu N.J., Zhou T. (2016). Free Energy Perturbation Calculation of Relative Binding Free Energy between Broadly Neutralizing Antibodies and the gp120 Glycoprotein of HIV-1. J. Mol. Biol..

[35] Pancera M., Shahzad-ul-Hussan S., Doria-Rose N.A., McLellan J.S., Bailer R.T., Dai K., Loesgen S., Louder M.K., Staupe R.P., Yang Y. (2013). Structural basis for diverse *N*-glycan recognition by HIV-1–neutralizing V1–V2–directed antibody PG16. Nat. Struct. Mol. Biol..

[36] Alexandre K.B., Gray E.S., Lambson B.E., Moore P.L., Choge I.A., Mlisana K., Karim S.S.A., McMahon J., O’Keefe B., Chikwamba R. (2010). Mannose-rich glycosylation patterns on HIV-1 subtype C gp120 and sensitivity to the lectins, Griffithsin, Cyanovirin-N and Scytovirin. Virology.

[37] Akkouh O., Ng T.B., Singh S.S., Yin C., Dan X., Chan Y.S., Pan W., Cheung R.C.F. (2015). Lectins with anti-HIV activity: A review. Molecules.

[38] Pang W., Tam S.-C., Zheng Y.-T. (2009). Current peptide HIV type-1 fusion inhibitors. Antivir. Chem. Chemother..

[39] Zhao B., Mankowski M.K., Snyder B.A., Ptak R.G., Liwang P.J. (2011). Highly potent chimeric inhibitors targeting two steps of HIV cell entry. J. Biol. Chem..

[40] Kagiampakis I., Gharibi A., Mankowski M.K., Snyder B.A., Ptak R.G., Alatas K., LiWang P.J. (2011). Potent Strategy To Inhibit HIV-1 by Binding both gp120 and gp41. Antimicrob. Agents Chemother..

[41] Qin L., Kufareva I., Holden L.G., Wang C., Zheng Y., Zhao C., Fenalti G., Wu H., Han G.W., Cherezov V. (2015). Crystal structure of the chemokine receptor CXCR4 in complex with a viral chemokine. Science.

[42] Zhang L., Herrera C., Coburn J., Olejniczak N., Ziprin P., Kaplan D.L., LiWang P.J. (2017). Stabilization and sustained release of HIV inhibitors by encapsulation in silk fibroin disks. ACS Biomater. Sci. Eng..

[43] Nedellec R., Coetzer M., Lederman M.M., Offord R.E., Hartley O., Mosier D.E. (2011). Resistance to the CCR5 inhibitor 5P12-RANTES requires a difficult evolution from CCR5 to CXCR4 Coreceptor use. PLoS ONE.

[44] Paul D., Boyer E.G., Krebs D.S.S. (1970). The Enzymes.

[45] Chang Y.-G., Kuo N.-W., Tseng R., LiWang A. (2011). Flexibility of the C-terminal, or CII, ring of KaiC governs the rhythm of the circadian clock of cyanobacteria. Proc. Natl. Acad. Sci. USA.

[46] Wishart D.S., Bigam C.G., Yao J., Abildgaard F., Dyson H.J., Oldfield E., Markley J.L., Sykes B.D. (1995). 1 H, 13 C and 15 N chemical shift referencing in biomolecular NMR. J. Biomol. NMR.

[47] Delaglio F., Grzesiek S., Vuister G.W., Zhu G., Pfeifer J., Bax A. (1995). NMRPipe: A multidimensional spectral processing system based on UNIX pipes. J. Biomol. NMR.

[48] Garrett D.S., Powers R., Gronenborn A.M., Clore G.M. (1991). A common sense approach to peak picking in two-, three-, and four-dimensional spectra using automatic computer analysis of contour diagrams. J. Magn. Reson..

[49] Laurence J.S., Blanpain C., Burgner J.W., Parmentier M., LiWang P.J. (2000). CC chemokine MIP-1β can function as a monomer and depends on Phe13 for receptor binding. Biochemistry.

[50] Li M., Gao F., Mascola J.R., Stamatatos L., Polonis V.R., Koutsoukos M., Voss G., Goepfert P., Gilbert P., Greene K.M. (2005). Human Immunodeficiency Virus Type 1 env Clones from Acute and Early Subtype B Infections for Standardized Assessments of Vaccine-Elicited Neutralizing Antibodies. J. Virol..

[51] Derdeyn C.A., Decker J.M., Bibollet-Ruche F., Mokili J.L., Muldoon M., Denham S.A., Heil M.L., Kasolo F., Musonda R., Hahn B.H. (2004). Envelope-constrained neutralization-sensitive HIV-1 after heterosexual transmission. Science.

[52] Wei X., Decker J.M., Liu H., Zhang Z., Arani R.B., Kilby J.M., Saag M.S., Shaw G.M., Kappes J.C., Wu X. (2002). Emergence of Resistant Human Immunodeficiency Virus Type 1 in Patients Receiving Fusion Inhibitor (T-20) Monotherapy Emergence of Resistant Human Immunodeficiency Virus Type 1 in Patients Receiving Fusion Inhibitor (T-20) Monotherapy. Antimicrob. Agents Chemother..

[53] Wei X., Decker J.M., Wang S., Hui H., Kappes J.C., Wu X., Salazar-Gonzalez J.F., Salazar M.G., Kilby J.M., Saag M.S. (2003). Antibody neutralization and escape by HIV-1. Nature.

[54] Jin H., Shen X., Baggett B.R., Kong X., LiWang P.J. (2007). The human CC chemokine MIP-1β dimer is not competent to bind to the CCR5 receptor. J. Biol. Chem..

