# Supplementary material for: The Effect of N-Terminal Cyclization on the Function of the HIV Entry Inhibitor 5P12-RANTES
Source: Int J Mol Sci. 2017 Jul 20;18(7):1575. doi: 10.3390/ijms18071575 (PMC5536063; doi:10.3390/ijms18071575)
Supplement: Supplementary file 1 [file ijms-18-01575-s001.pdf]

The effect of N-terminal cyclization on the function of the HIV entry inhibitor 5P12-RANTES

Anna F. Nguyen, Megan S. Schill, Mike Jian, and Patricia J. LiWang*

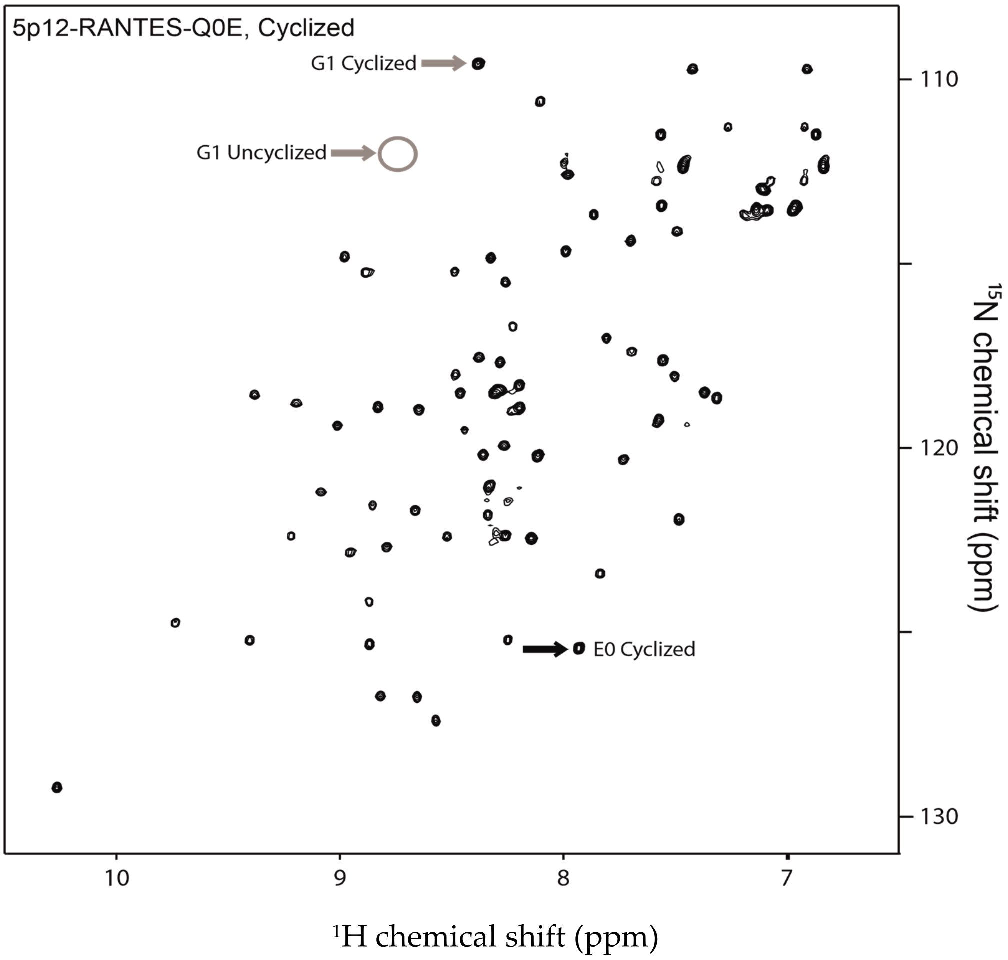

Supplementary Figure S1. HSQC spectrum of cyclized ^{15}N-labeled 5P12-RANTES-Q0E after being incubated at 50°C for 50 Days at pH 2.8; used for functional assays. NMR was performed in 20 mM sodium phosphate at pH 2.8, 25°C. N-terminal cyclization of E0 results in a shift of the G1 residue (grey arrows), as well as an appearance of the N-terminal pyroglutamate residue (lower black arrow), E0. This is in the same position as the peak corresponding to cyclized Q0 in 5P12-RANTES (Figure. 3A).

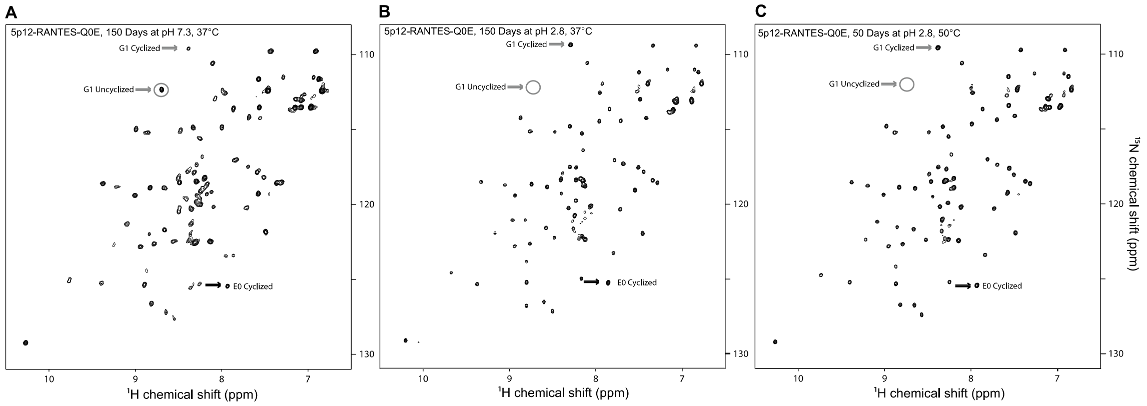

Supplemental Figure S2. HSQC spectra of ^{15}N-labeled Q0E, after varying incubation conditions. All spectra were measured at pH 2.8. **A**) 150 days incubation at pH7.3, 37°C; NMR measured at 25°C; **B**) 150 days incubation at pH 2.8, 37°C; NMR measured at 37°C; **C**) 50 day incubation at 50°C; NMR measured at 25°C. NMR was carried out in 20 mM sodium phosphate at pH 2.8. Cyclization results in a shift of the G1 residue (grey arrows), as well as an appearance of the N-terminal pyroglutamate residue (lower black arrow), E0. Figure B shows slight deviation in peaks due to temperature at which NMR was measured.

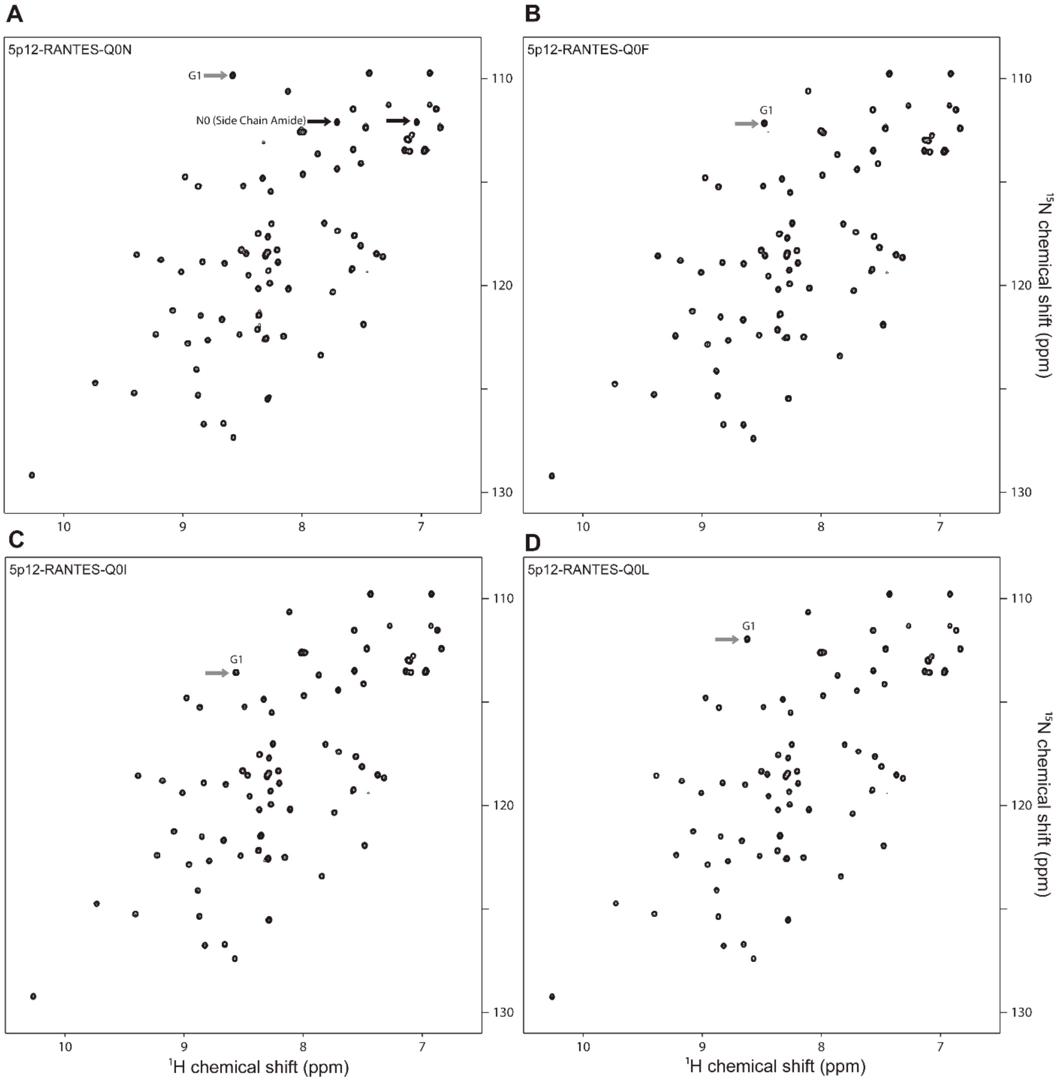

Supplemental Figure S3. HSQC spectra of ^{15}N-labeled **A**) 5P12-RANTES-Q0N, **B**) 5P12-RANTES-Q0F, **C**) 5P12-RANTES-Q0I, and **D**) 5P12-RANTES-Q0L, (pH 2.8, 20 mM sodium phosphate buffer, 25°C). The spectra are essentially identical to the 5P12-RANTES spectrum (Figure 2) except for a slight G1 shift (grey arrows) and a difference in the amide side chain peaks. B, C, and D do not have that amide side chain. A shows a slight shift in the amide side chain peaks that correspond to the Asn-0 NH2 group (black arrows). While N-terminal Glu and Gln can cyclize, Asn does not favorably cyclize due to the shorter side chain.

```
RANTES/CCL5:  -SPYSSDTTPCCFAYIARPLPRAHIKEYFYTSGKCSNPAVVFVTRKNRQVCANPEKKWVREYINSLEMS
                        ||||||||||||||||||||||||||||||||||||||||||||||||||||||||||
5P12-RANTES:  QGPPLMATQSCCFAYIARPLPRAHIKEYFYTSGKCSNPAVVFVTRKNRQVCANPEKKWVREYINSLEMS
```

Supplemental Figure S4. Sequence comparison of wild type RANTES/CCL5 and the variant 5P12-RANTES. The differences between the N-termini of the proteins are highlighted in bold.

Supplemental Table S1. CCR5 Signaling Activity (Fluorescence)	
5P12 Variant	*CCR5 signaling activity (% Max)*
RANTES, Wild Type	100%
5P12-RANTES Uncyclized	−0.4 ± 0.3 %
5P12-RANTES Cyclized	0.3 ± 0.2%
5P12-RANTES-Q0E Uncyclized	6.5 ± 0.6%
5P12-RANTES-Q0E Cyclized	−0.8 ± 0.2%
5P12-RANTES-Q0N	2.3 ± 0.5%
5P12-RANTES-Q0I	4.1±0.8%
5P12-RANTES-Q0F	5.0 ± 0.1%
5P12-RANTES-Q0L	−0.4 ± 1.5%

Supplementary Table S1. CCR5 signaling activity of 5P12-RANTES variants based on fluorescence assay using Fluo-4 AM and HeLa-5PL cells. Percent activity was determined by comparing to the maximal signal of positive control, wild type RANTES. Assays were performed in triplicate and repeated three times, at 300 nM concentration
